# Parametric simulation dataset of a 2.4 GHz patch antenna with slot for AI-based S11 prediction

**DOI:** 10.1016/j.dib.2025.112398

**Published:** 2025-12-17

**Authors:** Ameni Mersani, Kawther Mekki, Omrane Necibi

**Affiliations:** a University of Tunis El Manar, Faculty of Sciences Tunisia, Microwave Electronics Research Laboratory LR18ES43, 2092 Tunis, Tunisia; b ESPRIT M2M team, ESPRIT School of Engineering, Tunis, Tunisia

**Keywords:** Microstrip structures, Return loss modeling, Frequency-domain analysis, Parametric design sweep, Electromagnetic performance prediction, AI-driven RF component analysis, Machine learning

## Abstract

This dataset comprises over 55,000 simulation samples of a microstrip patch antenna engineered for operation near the 2.4 GHz frequency band—a key spectrum for wireless communication and IoT applications. Each sample records S11 reflection coefficient values (in decibels), mapping the return loss across a broad range of geometric configurations. Data were generated over a one-month period using parameter sweeps and variation studies with CST Microwave Studio, ensuring comprehensive coverage of design possibilities. The resulting dataset serves as a substantial resource for developing and benchmarking machine learning models in antenna performance prediction, design automation, and optimization tasks including impedance matching, bandwidth enhancement, and geometric optimization in RF and microwave engineering. To promote generalizability and practical relevance for the research community, future dataset releases will incorporate experimental validation and expand target parameters to include antenna impedance, thereby improving the robustness and utility for advanced AI-driven modeling and optimization.

Specifications Table

**Table:** 

Subject	Engineering & Materials science
Specific subject area	Microwave Engineering, Electromagnetic Design, Parametric Simulation, return loss prediction.
Type of data	CSV file (numerical data).
Data collection	The data were collected through electromagnetic simulations using CST Microwave Studio (Dassault Systems, version 2018). A microstrip patch antenna model was parametrically varied to cover a wide range of geometric configurations. Simulations computed the S11 reflection coefficient (in dB) around 2.4 GHz. Inclusion criteria focused on physically realizable antenna dimensions for IoT applications. No experimental measurements were involved. Data normalization involved scaling geometric parameters to a [0,1] range to facilitate machine learning model training.
Data source location	University of Tunis El Manar, Faculty of Sciences, Microwave Electronics Research Laboratory LR18ES43, Tunisia.
Data accessibility	Repository name: zenodo Data identification number: 10.5281/zenodo.15866821 Direct URL to data: https://zenodo.org/records/15866865
Related research article	https://indjst.org/articles/ai-powered-s11-prediction-for-a-compact-24-ghz-patch-antenna

## 1 Value of the Data

• Purpose and research problem: This dataset was created to accelerate and enhance microstrip antenna design through machine learning approaches, particularly focusing on S11 prediction—a key performance metric for RF engineers. By offering a comprehensive parametric sweep across thousands of geometric variations centered on the 2.4 GHz band, it addresses the need for large, high-quality data to drive ML-based optimization and design automation in wireless and IoT contexts. The real-world applicability and robustness of this resource have been validated through a peer-reviewed publication, which successfully employed this dataset for both training and evaluating S11 prediction models for compact textile antennas.
• Realistic, diverse simulation coverage:
• The dataset comprises over 55,000 simulated samples capturing wide-ranging geometric configurations, ensuring broad coverage of realistic design scenarios relevant to modern antenna challenges. Generated using advanced full-wave electromagnetic solvers, the data enables users to replicate and study practical phenomena encountered in RF and wearable antenna engineering [1].
• Comprehensive annotation for learning and benchmarking:
• Each sample includes detailed S11 values under varying parametric conditions, allowing nuanced analysis and interpretability for machine learning tasks. The structured annotation supports comparative analysis and fair benchmarking for algorithmic development, benchmarking, and cross-validation against experimental results.
• Enables a range of research and engineering tasks:
• The dataset is ideally suited for academic researchers and engineers working on RF, IoT, and antenna optimization. It supports model development for deep learning-based antenna design, comparative evaluation of ML architectures, rapid prototyping, and cost/time reduction versus traditional simulation workflows.
• Standardization and practical impact:
• Delivered in a standardized, easily accessible format, the dataset empowers reproducible research, robust benchmarking, and practical machine learning workflow integration. Its proven value is evidenced by the successful publication leveraging this data, demonstrating its immediate relevance and facilitating further innovation in electromagnetic and AI-assisted antenna development.

## 2 Background

To address the significant temporal and computational challenges associated with conventional parametric modeling in patch antenna design, this dataset was meticulously developed as a comprehensive and scalable resource for accelerating innovation in the field. Comprising 55,053 simulated samples of a microstrip patch antenna designed for operation at 2.4 GHz—an essential frequency band for modern IoT applications—the dataset captures a rich diversity of antenna geometries and electrical properties. Each entry is rigorously labeled with the relevant geometric characteristics and electrical parameters, with the primary target output being the S11 reflection coefficient, a critical measure of antenna matching and efficiency.

The central goal behind assembling this dataset was to empower the application of advanced machine learning frameworks for precise RF performance prediction and inverse antenna design. By providing exhaustive parametric coverage, the dataset enables detailed analysis of how specific geometric and material variations influence the reflection coefficient, allowing for smarter and faster design cycles. This not only dramatically reduces the time and computational resources traditionally required for antenna optimization but also opens new pathways for data-driven automation and intelligent model-based design in antenna engineering [2].

Beyond serving as a valuable reference for developing and benchmarking AI-driven models, the dataset acts as a catalyst for reproducible and scalable experimentation. Its structured organization ensures ease of integration into various machine learning pipelines, fostering fair comparative analyses and standardized model assessment across the research community. Furthermore, the practical relevance of the dataset has already been validated by its pivotal role in a peer-reviewed research article, wherein it supported the development and evaluation of machine learning algorithms for S11 prediction in compact textile antenna structures. This demonstrated use case not only highlights its immediate impact but also underscores its broader potential for facilitating rapid, reliable, and innovative advances in RF, IoT, and wearable antenna technologies [[3], [4]].

The remainder of this manuscript is structured as follows. Section 1 presents the objective of the dataset and summarizes its main contributions to the field of antenna design, optimization, and intelligent RF engineering. Section 2 delivers a comprehensive description of the dataset, including its structure, key features, and details on parameter labeling and file format. Section 3 outlines the experimental design, simulation environment, and methodological steps employed in generating the dataset, and is subdivided into three parts: Data Generation, describing the simulation setup and parameter sweep strategy; Data Preprocessing, explaining data validation, cleaning, and structuring procedures; and Documentation, which details the codebook creation, metadata, and supporting materials to ensure reproducibility and ease of use.

## 3 Data Description

The dataset encompasses a comprehensive collection of 55,053 simulated samples, specifically constructed to advance machine-learning applications in microstrip antenna design for wireless and IoT technologies. Each sample captures the electromagnetic properties of a unique microstrip patch antenna structure, with an emphasis on the reflection coefficient (S11, in dB) evaluated primarily at 2.4 GHz.

The core of the dataset consists of systematically generated simulation data, where each row represents a distinct antenna design defined by a set of 12 input features. These features describe key geometric and material properties including the width and length of the substrate, patch, and feed line; the height and width of the radiating patch; the dimensions of slots in the patch; and substrate thickness, as summarized in Table 1. The only output variable is the S11 value, which quantifies the antenna’s impedance matching, providing direct insight into its performance at the designated frequency.

**Table 1:** Content of the related field dataset (DOI 10.5281/zenodo.15866821).

Column Name	Description	Unit
width_substrate “w”	Width of the dielectric substrate	mm
width_imp_line “wf”	Width of the microstrip feed line	mm
length_imp_line “Lf”	Length of the microstrip feed line	mm
substrate_height “h”	Thickness of the substrate	mm
patch_height “z”	Height of the radiating patch	mm
width_slot “Ws”	Width of the slot cut in the patch	mm
length_slot “Ls”	Length of the slot cut in the patch	mm
patch_width “Wp”	Width of the patch	mm
substrate_length “L”	Length of the substrate	mm
patch_length “Lp”	Length of the patch	mm
Frequency	Frequency at which S11 was evaluated (typically 2.4 GHz)	GHz
S11	Reflection coefficient (S11) at the given frequency	dB

All simulations were performed in CST Microwave Studio®, using automated parameter sweeps to systematically vary geometric and material characteristics across practical ranges for IoT-centric microstrip patch antennas. Parameter ranges were chosen to capture realistic design diversity applicable to wearable and connected device engineering, ensuring that the dataset encompasses the full spectrum of common form factors, dielectric properties, and slot configurations relevant to modern RF applications.

For each design, the dataset reports:

• Substrate and patch dimensions (length, width, height, and thickness)
• Slot and feed line characteristics
• Material properties, as reflected in the geometry and layer parameters
• The exact frequency used for S11 evaluation (primarily 2.4 GHz, though some samples cover 1.8–2.8 GHz for broader spectrum studies)
• The target S11 (in dB), representing the simulated return loss at the specified frequency

This structure enables the dataset to serve not only as a resource for regression and performance prediction tasks, but also for more advanced machine learning applications: surrogate modeling, sensitivity analyses, design space exploration, and data-driven inverse design for target S11 specifications.

The dataset is delivered as a single, clean CSV file with no missing values, ensuring easy adoption for data science workflows in Python, MATLAB, R, or Excel. A detailed codebook is provided, describing each field, parameter range, and units, alongside a snapshot figure illustrating the antenna geometry and simulation model.

The accompanying repository on Zenodo organizes the content for maximum clarity and reproducibility. The main dataset file (Ant_ML_Dataset.csv) contains all samples, while supplementary files include the codebook (CODEBOOK.md), a subset of representative design images, and scripts for data loading and preprocessing. All files are structured for immediate integration with popular machine learning frameworks, allowing users to quickly set up their own regression, optimization, or benchmarking tasks.

To facilitate further research and reproducibility, the dataset also provides:

• Fixed train/validation/test splits supporting standardized benchmarking of ML algorithms
• A complete README fleshing out the simulation process, preprocessing pipeline, and any data cleaning steps performed
• Guidance on extending the dataset with experimental measurements in future releases, to enable experimental-ML hybrid workflows

The holistic organization, annotation, and diversity of this dataset make it a robust benchmark for the antenna research community, and an ideal resource for anyone pursuing data-driven electromagnetic optimization, performance prediction, or intelligent antenna design. An overview of the input and output variables, as well as their data types and units, is provided in Table 1.

This structured, annotated, and rigorously validated resource enables reproducible, state-of-the-art research at the intersection of antenna engineering and artificial intelligence.

**Fig. 1:**
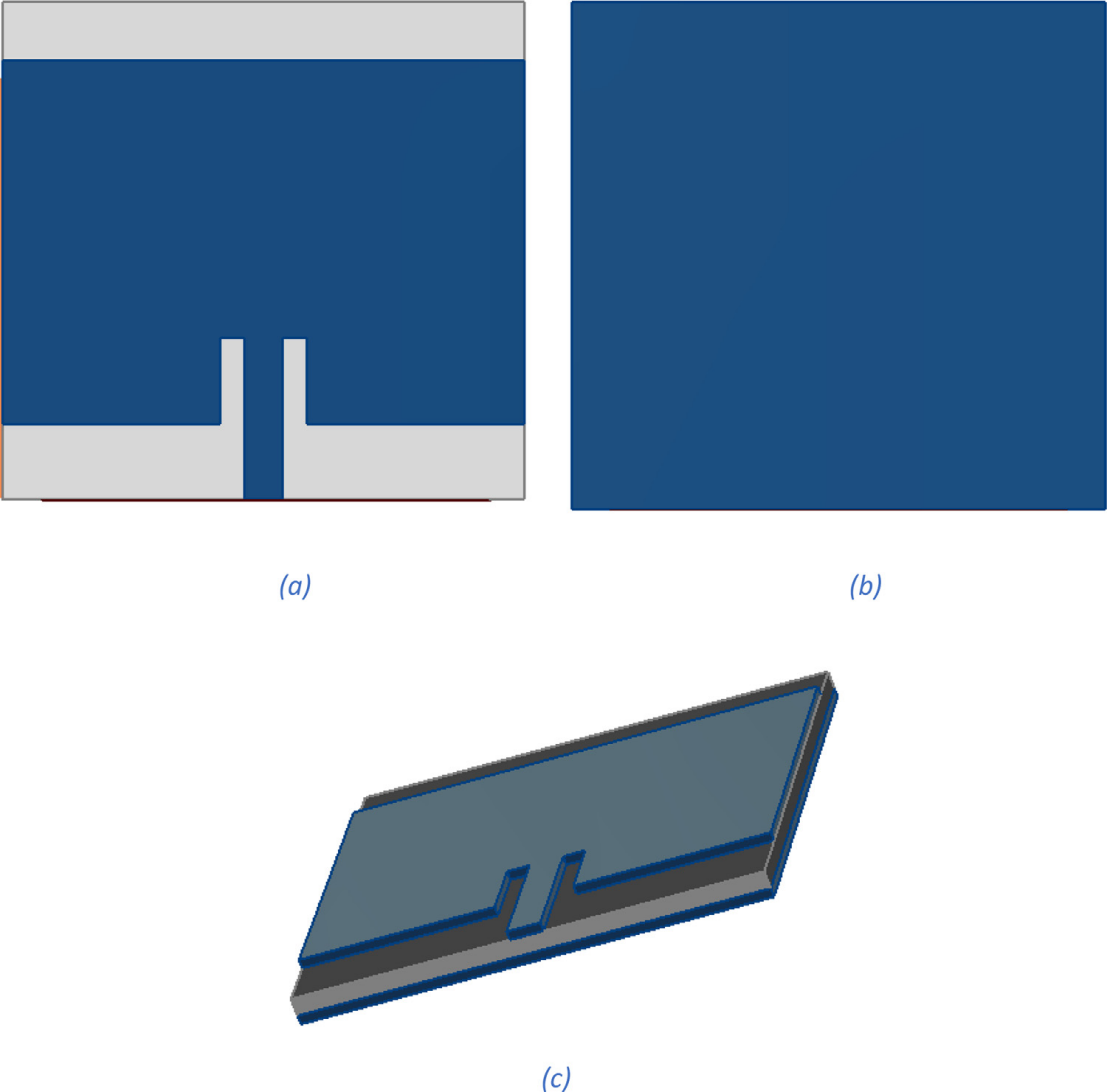
Antenna structure: (a) side view (b) back view (c) perspective view.

To provide clear physical context for the dataset parameters, Fig. 1 schematically illustrates the geometry of the simulated microstrip patch antenna, including the main dimensional features: substrate, patch, slot, and feed line. Each labeled dimension corresponds directly to an input variable in the dataset’s feature set (see Table 1). This representation facilitates the interpretation of how geometric variations influence the electromagnetic properties and performance metrics such as S11 [[1], [2], [3], [4], [5]].

The Zenodo record for the Patch Antenna Dataset is organized into four main components: All_Simulated_Data (the complete set of 55,053 antenna simulation samples in CSV/XLS format), Machine_Learning_Models (preprocessing scripts, model training notebooks, and saved model files), Model_Evaluation_and_Results (validation metrics, feature importance plots, correlation matrices, and visualizations of predicted versus actual S11), and Documentation (including the main README, codebook, and parameter descriptions).

The All_Simulated_Data folder contains the antenna geometry dataset with detailed input parameters (patch, substrate, slot, and feed line dimensions), corresponding to each design’s S11 target output. The Machine_Learning_Models component provides Jupyter notebooks and scripts for data preprocessing and model training, covering linear regression, support vector regression, random forest, XGBoost, and k-nearest neighbors; saved model files are available for reproducibility. Within Model_Evaluation_and_Results, users will find exported metrics in CSV and PNG format, feature importance visualizations, comparative plots of actual versus predicted S11, and Pearson correlation matrices for feature analysis.

**Fig. 2:**
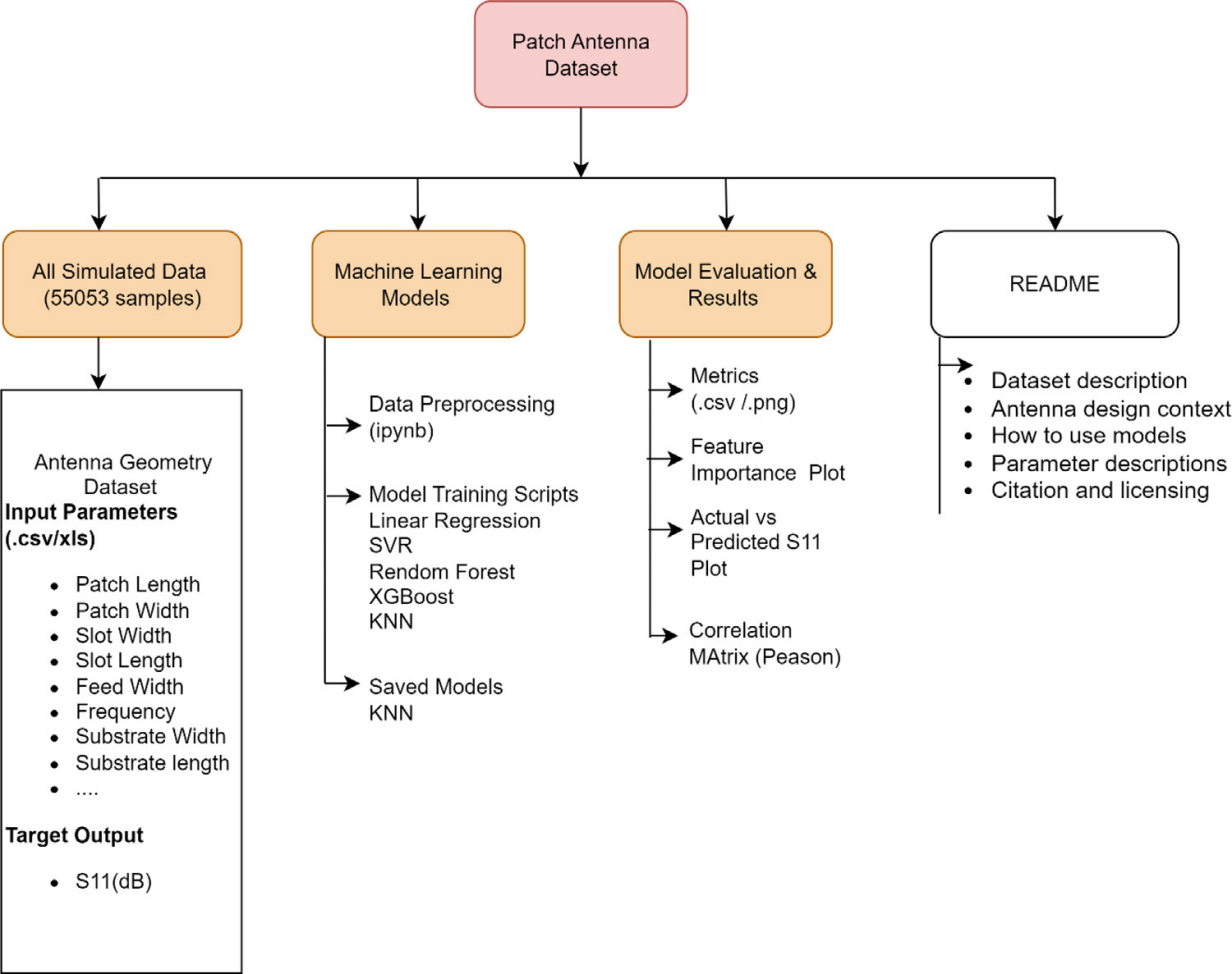
Overview of the dataset structure and processing workflow.

The Documentation folder features a comprehensive README detailing the dataset context, usage instructions, parameter definitions, licensing and citation information. Each main folder is equipped with its own readme file, outlining its content, recommended usage, and specific notes for integrating the dataset with external machine learning pipelines. This modular and standardized structure facilitates straightforward dataset adoption, reproducible experimentation, and transparent benchmarking for RF and antenna data science applications. An overview of the full directory layout is shown in Fig. 2.

## 4 Experimental Design, Materials and Methods

### 4.1 Workflow summary

**Fig. 3:**
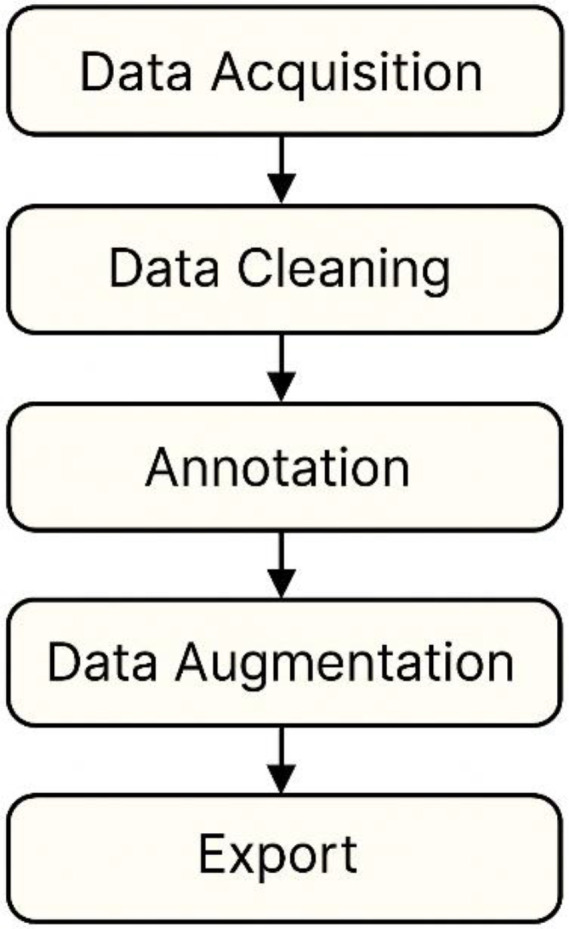
Workflow.

The dataset creation workflow (see Fig. 3) consists of a structured sequence of steps tailored for efficient and reproducible RF simulation and data-driven machine learning research: (1) Data Generation, where patch antenna geometries and material parameters were systematically varied and simulated using CST Microwave Studio®; (2) Data Cleaning, in which the simulation outputs were inspected to remove outliers or erroneous runs and to ensure parameter consistency; (3) Data Aggregation and Structuring, converting raw simulation results into standardized CSV/XLS files with labeled features and clear documentation; (4) Data Augmentation, expanding the variability of the dataset through additional parametric sweeps and controlled randomization within practical physical constraints; and (5) Export and Annotation, preparing curated datasets with accompanying codebooks, usage guides, and README files to enable reproducibility and integration into machine learning workflows.

### 4.2 Data generation

Patch antenna samples were simulated primarily for the 2.4 GHz frequency band—a pivotal spectrum for IoT and wireless applications. Using CST Microwave Studio®, parametric sweeps were performed by varying key geometric attributes (patch and slot dimensions, substrate thickness, feed line lengths/widths), ensuring realistic diversity in form factors and material composition. Each simulated antenna was assigned to a unique identifier, and its input parameters were logged along with the computed S11 value at the designated frequency. This approach enabled coverage of 55,053 unique antenna configurations, capturing an extensive range of electromagnetic behaviors.

### 4.3 Simulation materials and parameters

All simulations were carried out using a standard workflow in CST Microwave Studio® on high-performance computing infrastructure. Materials for substrate and patch elements were selected according to common dielectric properties used in wearable and IoT antenna research. Dimensions for patches, slots, and feed lines were parameterized over practical intervals determined by literature benchmarks and prior design experience. The simulation setup was validated with benchmark models to ensure consistency.

### 4.4 Data cleaning and validation

Post-simulation, the dataset was rigorously inspected for errors such as incomplete runs, physically implausible geometries, and duplicate samples. Outlier S11 values and inconsistent parameterizations were flagged and removed. The principal investigator conducted visual and statistical inspections to ensure high-quality, representative samples across the design space.

### 4.5 Data aggregation and structuring

Cleaned simulation results were exported to CSV and XLS formats, with each row corresponding to a unique antenna design defined by 12 labeled input features and one output (S11, in dB). A detailed codebook was created to document parameter definitions, units, and value ranges. Supplementary reference images of representative antenna geometries were captured and included for context.

### 4.6 Data augmentation

To further expand the dataset and support robust ML model training, additional parametric sweeps were generated by randomizing input values within allowable intervals. Sampling strategies were carefully controlled to maintain physical plausibility and coverage of the design space. These augmentations mirror typical design variations encountered in practical antenna engineering.

### 4.7 Annotation and export

The final dataset includes all input features, simulation frequency, and output S11 value, presented as a single CSV file for interoperability with Python, MATLAB, R, and Excel platforms. Accompanying documentation consists of a main README, codebook, and usage notes for integrating the dataset into regression, surrogate modeling, or optimization workflows.

Sample scripts and usage guides are provided for loading and preprocessing the data, with example Jupyter notebooks to facilitate reproducible experimentation and fair benchmarking. The dataset is accessible via Zenodo and includes guidance for extending and reusing simulation scripts for additional antenna configurations or experimental validation.

This comprehensive and annotated dataset serves as a foundation for advancing machine learning-driven antenna research, enabling users to develop, benchmark, and deploy intelligent RF design solutions with reproducibility and scalability in mind.

The structure and organization of the dataset were deliberately designed to enable future extensions and to support expanding research needs in antenna engineering and machine learning. Beyond the current simulated data for S11 prediction, ongoing work involves the physical fabrication and experimental measurement of selected antenna prototypes. This will allow integration of real-world measurement results, thereby strengthening the dataset's value for experimental validation and hybrid data-driven workflows.

In forthcoming updates, additional features such as measured gain and directivity will be included, providing users with richer labels for advanced model development and comprehensive performance analysis. The dataset is also structured to easily accommodate new geometric configurations and to incorporate additional environmental and material parameters, such as the effects of humidity, substrate type (including textile and flexible materials), and bending or deformation scenarios. These extensions will further enhance the dataset's versatility, enabling the study and optimization of antennas for challenging use-cases in IoT, wearable, and flexible electronics applications. By maintaining a modular and extensible format, the dataset aims to serve as a living resource for the community, continually evolving to address emerging research questions and technological advancements.

## Limitations

Not applicable.

## Ethics Statement

This work complies with the ethical standards required for publication in Data in Brief (Elsevier Policies and Ethics).

No human participants or animal subjects were involved in this study. All data were generated through numerical simulations and do not contain any personally identifiable or sensitive information.

## Credit Author Statement

**Ameni Mersani:** Conceptualization, Writing – original draft, Writing, Methodology, Software; **Kawther Makki:** Writing, Reviewing draft preparation; **Omrane Necibi:** Writing- Reviewing and Editing.

## Data Availability

ZenodoDataSet (Original data).

## Appendix Supplementary materials

Image, application 1
