# Supplementary material for: Parametric simulation dataset of a 2.4 GHz patch antenna with slot for AI-based S11 prediction
Source: Data Brief. 2025 Dec 17;64:112398. doi: 10.1016/j.dib.2025.112398 (PMC12830091; doi:10.1016/j.dib.2025.112398)
Supplement: Supplementary file 1 [file mmc1.zip › Patch Antenna.pdf]

Data in Brief
Open access
Article template

ARTICLE INFORMATION

Article title

Parametric Simulation Dataset of a 2.4 GHz Patch Antenna with Slot for AI-based S11 Prediction

Authors

Ameni Mersani, Kawther Makki, Omran Ncibi*

Affiliations

University of Tunis El Manar, Faculty of Sciences Tunisia, Microwave Electronics Research Laboratory LR18ES43, 2092 Tunis, Tunisia; Department of Computer Engineering, College of Computer Science and Engineering, University of Ha'il, Ha'il 2440, Saudi Arabia; Department of Computer Science, College of Arts and Sciences at Tabarjal, Jouf University, Jouf 72388, Saudi Arabia.

Corresponding author's email address and Twitter handle

Ameni.mersani@fst.utm.tn

Keywords

Microstrip structures, Return loss modeling, Frequency-domain analysis, Parametric design sweep, Electromagnetic performance prediction, AI-driven RF component analysis.

Abstract

This dataset contains more than 55,000 simulation samples of a microstrip patch antenna specifically designed to operate around the 2.4 GHz frequency band, which is widely used in wireless communication and IoT applications. Each sample includes the S11 reflection coefficient values (in decibels), representing the return loss for various geometric configurations of the antennaSimulations were run with CST Microwave Studio over the course of one month, with a large variation and parameter sweeping. This extensive dataset is well-suited for training and testing machine learning models for antenna performance prediction, design automation, and optimization tasks such as impedance matching, bandwidth maximization, and structure optimization in RF and microwave engineering.

SPECIFICATIONS TABLE

[]

**Subject**	Engineering & Materials science
**Specific subject area**	*Microwave Engineering, Electromagnetic Design, Parametric Simulation, return loss prediction.*
**Type of data**	*CSV file (numerical data).*

**Data collection**	*The data were collected through electromagnetic simulations using CST Microwave Studio (Dassault Systems, version 2018). A microstrip patch antenna model was parametrically varied to cover a wide range of geometric configurations. Simulations computed the S11 reflection coefficient (in dB) around 2.4 GHz. Inclusion criteria focused on physically realizable antenna dimensions for IoT applications. No experimental measurements were involved. Data normalization involved scaling geometric parameters to a [0,1] range to facilitate machine learning model training.*
**Data source location**	*University of Tunis El Manar, Faculty of Sciences, Microwave Electronics Research Laboratory LR18ES43, Tunisia.*
**Data accessibility**	Repository name: zenodo<br>Data identification number: 10.5281/zenodo.15866821<br>Direct URL to data: **https://zenodo.org/records/15866865**
**Related research article**	*None*

VALUE OF THE DATA

- Enables machine learning models to predict S11 for microstrip antennas.
- Helps reduce the cost and time associated with full-wave simulations.
- Useful for academic researchers and engineers working in RF, IoT, and antenna optimization.
- Facilitates comparative analysis and model benchmarking.
- Serves as a realistic training dataset for deep learning in antenna design.

BACKGROUND

To overcome the temporal and computational constraints of traditional parametric models in patch antenna design, the dataset was formed. The dataset consists of 55,053 samples simulated for a 2.4 GHz patch antenna intended for IoT applications, each labeled with geometric and electrical parameters, whose target output is the reflection coefficient, S11. The objective was to allow machine learning frameworks to be applied for exact prediction of performance and inverse design, thus shortening the antenna design time. The dataset supports the development and benchmarking of AI-based models and acts as a starting point for data-driven optimization of antenna engineering. It supports the parallel research article by providing a structured and scalable dataset that facilitates reproducible experimentation and model assessment.

DATA DESCRIPTION

The dataset contains 12 features and 1 target variable:

- *Input parameters: Widths, lengths, and thicknesses of substrate, patch, slots, and feed line.*
- *Frequency in GHz: from 1.8 to 2.8 GHz.*
- *Output: S11 (in dB), representing the antenna's return loss.*

The file contains 55,053 rows with no missing values. The dataset is stored as a single CSV file and is accompanied by a codebook and a snapshot of the antenna design.

EXPERIMENTAL DESIGN, MATERIALS AND METHODS

Simulations were conducted using CST Microwave Studio's frequency domain solver. The antenna design was centered around a rectangular patch fed by a microstrip line, with two slots for impedance matching. Each parameter (slot length, width, patch dimensions, substrate height, etc.) was varied in a controlled sweep, and the corresponding S11 was extracted for each frequency point.

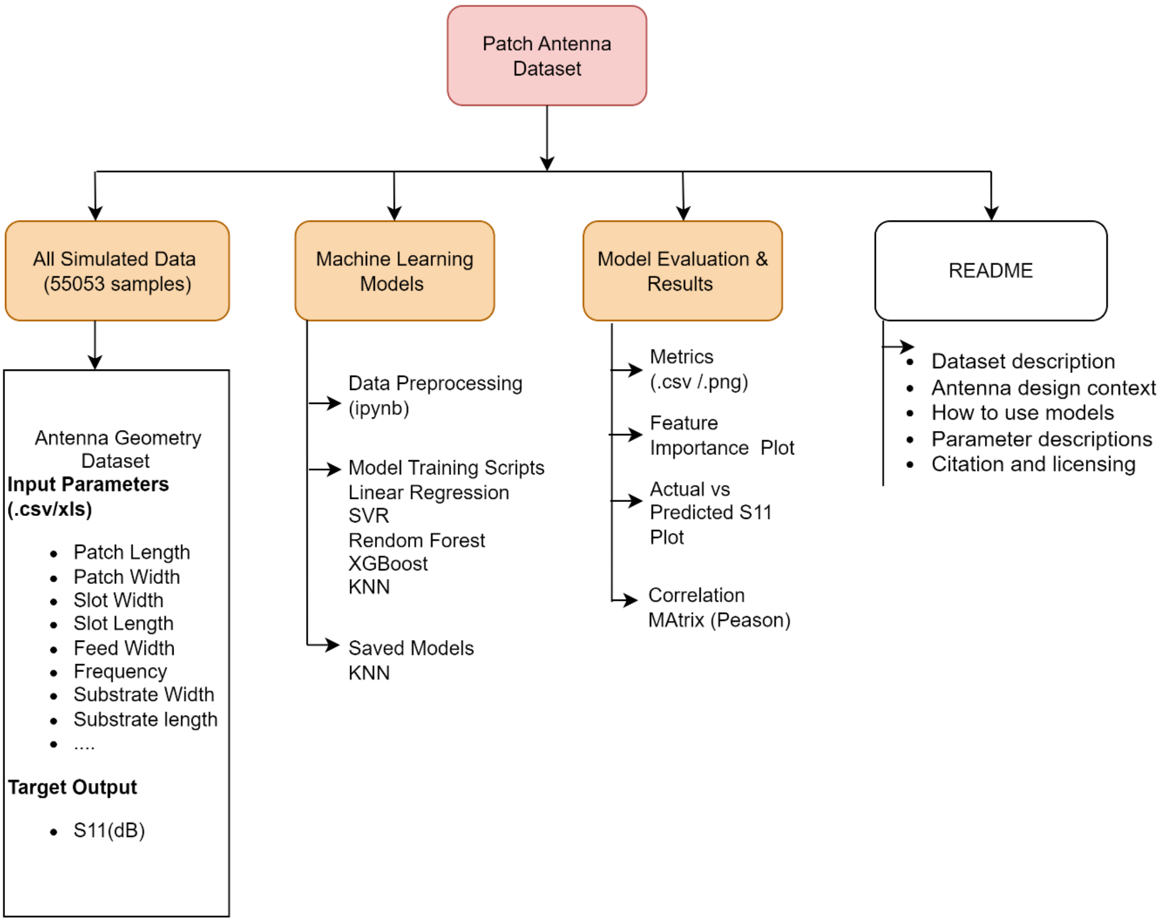

LIMITATIONS

Not applicable.

ETHICS STATEMENT

None

CRediT AUTHOR STATEMENT

Ameni Mersani: *Conceptualization, Methodology, Software.* ***Kawther Makki****: Writing, Original draft preparation.* ***Omran Ncibi:*** *Writing- Reviewing and Editing..*

ACKNOWLEDGEMENTS

This research did not receive any specific grant from funding agencies in the public, commercial, or not-for-profit sectors.

DECLARATION OF COMPETING INTERESTS

- The authors declare that they have no known competing financial interests or personal relationships that could have appeared to influence the work reported in this paper.

REFERENCES

[1] mersani, A. (2025). Simulated Patch Antenna for 2.4 GHz Applications [Data set]. Zenodo. https://doi.org/10.5281/zenodo.15866865

[2] Prottoy, S. S., Rana, M. M., & Alam, N. (2024). *A Meander Line Microstrip Patch Antenna for Internet of Things (IoT) Applications*. 605–608. https://doi.org/10.1109/peeiacon63629.2024.10800406

[3] Zambak, M. F., Al-Bawri, S. S., Jusoh, M., Rambe, A. H., Vettikalladi, H., Albishi, A. M., & Himdi, M. (2023). A Compact 2.4 GHz L-Shaped Microstrip Patch Antenna for ISM-Band Internet of Things (IoT) Applications. *Electronics*, *12*(9), 2149. https://doi.org/10.3390/electronics12092149

[4] Rana, Md. S., Islam, O., Shikha, S. A., & Faisal, M. (2023). *IoT Application using a Rectangular 2.4 GHz Microstrip Patch Antenna*. 1–4. https://doi.org/10.1109/ICONAT57137.2023.10080448
