# Supplementary figures and images for: Parametric simulation dataset of a 2.4 GHz patch antenna with slot for AI-based S11 prediction
Source: Data Brief. 2025 Dec 17;64:112398. doi: 10.1016/j.dib.2025.112398 (PMC12830091; doi:10.1016/j.dib.2025.112398)

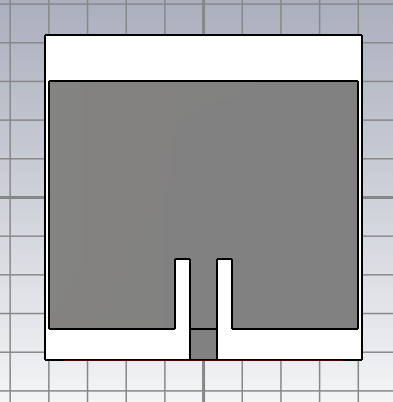

Supplement: Supplementary file 1 [file mmc1.zip › Antenna geometry.png]
